# 2538. The Impact of Rifampicin on Blood Pressure in Patients Receiving Nifedipine: A Retrospective Study

**DOI:** 10.1093/ofid/ofad500.2155

**Published:** 2023-11-27

**Authors:** Khalid Eljaaly

**Affiliations:** King Abdulaziz University, Saudi Arabia, Jeddah, Saudi Arabia

## Abstract

**Background:**

No comparative study has been published to date to evaluate the impact of drug-drug interaction (DDI) between rifampin (CYP3A4 inducer) and nifedipine on clinical outcomes. The aim of this study was to investigate the change in systolic blood pressure (SBP) after rifampin initiation in hypertensive patients who were receiving oral nifedipine.

**Methods:**

This retrospective cohort study was conducted at King Abdulaziz University Hospital. We included hospitalized adults ≥18 years who were started on rifampin while they were receiving oral nifedipine for hypertension. Patients with uncontrolled hypertension (baseline SBP of >140 mmHg) and those receiving beta-blockers were excluded. The study outcomes were SBP changes by day 8 after rifampin initiation, which were compared by Chi-squared test.

**Results:**

A total of 16 patients were included. The median maximum increase in SBP from baseline after starting rifampin was 24 [17.75 - 29] mmHg. The median SBP did not increase significantly on the second day (135 [128.5-141.5]; P=0.147) but the increases were significant on the following days. The median SBP was 146.5 [140.5-154.25] (P=0.002) on the third day, 142.5 [132.75-160.5] (P=0.009) on the fourth day, 149.5 [136-159.75] (P=0.005) on the fifth day, 146.5 [134.25-159.5] (P=0.012) on the sixth day, 149 [143.25-154.5] (P=0.007) on the seventh day, 151.5 [143.75-163] (P=0.007) on the eighth day.

**Conclusion:**

Initiation of rifampin in hypertensive nifedipine-treated patients was associated with a statistically and clinically significant increase in SBP starting from day 3 of rifampin. This supports the need to avoid nifedipine use in patients taking rifampin.

**Disclosures:**

**Khalid Eljaaly, PharmD, MS, BCPS, BCIDP, FCCP**, Reckitt Benckiser Healthcare International Ltd, UK: Member of the Global Respiratory Infection Partnership, supported by unrestricted educational grant from Reckitt Benckiser Healthcare Ltd

